# A simple classification of cranial–nasal–orbital communicating tumors that facilitate choice of surgical approaches: analysis of a series of 32 cases

**DOI:** 10.1007/s00405-016-4003-8

**Published:** 2016-03-26

**Authors:** Yue-fei Deng, Bing-xi Lei, Mei-guang Zheng, Yi-qing Zheng, Wei-liang Chen, Yu-qing Lan

**Affiliations:** Department of Neurosurgery, Center of Cranio-Maxillofacial Surgery, Sun Yat-Sen Memorial Hospital, Sun Yat-Sen University, 107 Yanjiang Road West, Guangzhou, 510120 China; Department of Otolaryngology, Center of Cranio-Maxillofacial Surgery, Sun Yat-Sen Memorial Hospital, Sun Yat-Sen University, Guangzhou, China; Department of Maxillofacial Surgery, Center of Cranio-Maxillofacial Surgery, Sun Yat-Sen Memorial Hospital, Sun Yat-Sen University, Guangzhou, China; Department of Ophthalmology, Center of Cranio-Maxillofacial Surgery, Sun Yat-Sen Memorial Hospital, Sun Yat-Sen University, Guangzhou, China

**Keywords:** Cranial–nasal–orbital region, Tumor, Classification, Surgical approach

## Abstract

Cranial–nasal–orbital communicating tumors involving the anterior and middle skull base are among the most challenging to treat surgically, with high rates of incomplete resection and surgical complications. Currently, there is no recognized classification of tumors with regard to the choice of surgical approaches. From January 2004 to January 2014, we classified 32 cranial–nasal–orbital communicating tumors treated in our center into three types according to the tumor body location, scope of extension and direction of invasion: lateral (type I), central (type II) and extensive (type III). This classification considerably facilitated the choice of surgical routes and significantly influenced the surgical time and amount of hemorrhage during operation. In addition, we emphasized the use of transnasal endoscopy for large and extensive tumors, individualized treatment strategies drafted by a group of multidisciplinary collaborators, and careful reconstruction of the skull base defects. Our treatment strategies achieved good surgical outcomes, with a high ratio of total resection (87.5 %, 28/32, including 16 cases of benign tumors and 12 cases of malignant tumors) and a low percentage of surgical complications (18.8 %, 6/32). Original symptoms were alleviated in 29 patients. The average KPS score improved from 81.25 % preoperatively to 91.25 % at 3 months after surgery. No serious perioperative complications occurred. During the follow-up of 3 years on average, four patients with malignant tumors died, including three who had subtotal resections. The 3-year survival rate of patients with malignant tumors was 78.6 %, and the overall 3-year survival rate was 87.5 %. Our data indicate that the simple classification method has practical significance in guiding the choice of surgical approaches for cranial–nasal–orbital communicating tumors and may be extended to other types of skull base tumors.

**Electronic supplementary material:**

The online version of this article (doi:10.1007/s00405-016-4003-8) contains supplementary material, which is available to authorized users.

## Introduction

Tumors in the anterior and middle skull base and those in the nasal cavity, sinuses and orbits frequently communicate and reciprocally invade [1–3]. These tumors are among the most challenging to treat surgically, with high rates of incomplete resection, surgical complications and sequelae owing to the involvement of functional structures, difficult access and the creation of large dural and bone defects after the removal of the tumor [1–3]. The rate of surgical complications in craniofacial tumor resections range from 33 to 50 % [4–6]. Selection of an appropriate surgical approach with respect to the best perspective of exposure, the shortest surgical distance and the lowest degree of brain tissue stretch is a crucial issue in skull base surgery. A literature review revealed more than 20 variations of surgical approaches, which can be summarized as transcranial, transfacial and a combined cranial–facial approach [7–10]. For cranial tumors located at the same location, there are two or three surgically possible approaches, and the one chosen varied greatly among different specialties [7–10]. For example, otolaryngologists may consider that the combined craniofacial approach is the standard for skull base tumors communicating with the nasal cavity [11]; however, neurosurgeons prefer the simple transcranial approach that decreases surgical time without affecting the efficacy and safety of surgery [8]. Several classification methods of skull base tumors based on the site of tumor origin and location or biological behavior (benign or malignant) are available [12]. Similarly, a number of classification methods have been adopted specifically for malignant ethmoidal tumors [13]. These classifications provide individualized guidance for specific types of tumors, but have not formed a recognized standard for the choice of surgical routes, particularly for cranial–nasal–orbital communicating tumors.

From January 2004 to January 2014, we used a simple classification method with regard to the location and direction of tumor invasion to classify 32 cases of such tumors treated in our center. This classification method greatly facilitated the choice of surgical routes. In addition, individualized strategies of tumor resection and skull base defect repair based on pre- and perioperative pathological findings were adopted by a group of multidisciplinary collaborators from neurosurgery, oral and maxillofacial surgery, otolaryngology and ophthalmology. The combined strategy achieved ideal treatment efficacy with low rates of complications and morbidity, and zero perioperative deaths.

## Materials and methods

The procedures of this study complied with the ethical criteria of the Human Study Committee of Sun Yat-Sen Memorial Hospital, Sun Yat-Sen University, Guangzhou, China, and the study was approved by the same committee. All patients agreed to the procedures and signed a written informed consent form.

*Inclusion criteria* From January 2004 to January 2014, our multidisciplinary collaborative group treated 32 patients with cranial–nasal–orbital tumors. All patients met the following criteria: ① 15–75 years old and Karnofsky score ≥70; ② no severe liver, kidney, lung, heart or other organ dysfunction; ③ no previous history of anterior-medial skull base tumor surgery or radiotherapy; ④ pre-surgical diagnosis confirmed by enhanced computed tomography (CT), and magnetic resonance imaging (MRI) showing that the scope of tumor invasion involved the nasopharynx, orbit, ethmoid and cranial cavity simultaneously; and ⑤ diagnosis of cranial–nasal–orbital communicating tumors confirmed by intraoperative findings and post-surgical histopathology.

*Exclusion criteria* Patients were excluded if they met the following criteria:① aged <15 years or >75 years; ② had a fever or had obvious inflammation in the nasopharyngeal skull; ③ had severe respiratory disease, heart disease, kidney disease or blood system diseases; ④ had a KPS score less than 70; ⑤ had a history of surgery or radiotherapy of the skull base tumors.

### Clinical data

The 32 patients included 20 males and 12 females with an average age of 46.3 years (range 16–75 years). The average duration of disease was 11.2 months (range 2 weeks to 6 years). The average Karnofsky score was 81.25, including 20 cases with a score of 80 and 8 cases with a score of 90. The pathological types of tumors are summarized in Table 1, and the detailed symptoms are summarized in Supplementary Table 1.

**Table 1:** Tumor types

Tumor type	Number of cases
Benign	
Neurilemmoma	4
Nasopharyngeal angiofibroma	3
Meningioma	5
Ossifying fibroma	2
Pituitary adenoma	1
Inverted papilloma	1
Paragangliocytoma	1
Malignant	
Esthesioneuroblastoma	5
Squamous cell carcinoma	3
Malignant fibrosarcoma	2
Lacrimal gland cell carcinoma	1
Melanoma	1
Transitional cell carcinoma	1
Small cell carcinoma	1
Transitional cell carcinoma	1

### Imaging examinations

All patients underwent CT and enhanced MRI before surgery and at 1 and 6 months after surgery. For tumors with a larger tumor body and abundant vascularization, 16 patients underwent magnetic resonance angiography (MRA), 12 underwent digital subtraction angiography (DSA) and 7 underwent external jugular vascular embolization for vessels supplying the tumors before surgery. There were 11 cases in which the main tumor body was located laterally and 21 cases in which it was bilateral. Seventeen cases exhibited expansion or disruption in the optic nerve foramen or superior orbital fissure, and destruction of the orbital roof, orbit or skull base bone was found in all 32 cases. The average diameter of the tumors was 5.7 cm (range 3–14.5 cm). In 12 cases, the diameter was <4 cm, and in 20 cases it was ≥4 cm. The average diameter of the skull base bone defects was 4.38 cm (range 3.3–7 cm).

The details about the tumor body location, extension/invasion and the affected tissues are listed in Supplementary Table 4. As shown by CT and MRI, the major part of tumor body in 15 cases was located at the skull base (SB): 12 at the anterior SB (3 left, 2 right, and 7 central SB), 1 at both the anterior and middle SB and 2 at the junction of the anterior and middle SB. In two patients, the major part of tumor body was located at the sella or sella and the petrocilivus. Thus, in 17 patients, the major part of tumors were located in the intracranial cavity. For 12 patients, the major part of tumor body was located extracranially, i.e., the orbits, nasopharynx and/or the nasal sinuses (ethmoid, sphenoid, subfrontal); for 3 patients, the major part of tumor body was located at both the SB and the orbits/nasal sinuses. The locations of the major part of tumor body are summarized in Supplementary Table 5.

### Tumor classification

Based on pre-surgical CT and MRI imaging and perioperative observation, tumors were classified according to the location of the main tumor body and the scope and direction of invasion (Table 2). The types were defined as follows:

**Table 2:** Summary of tumor classification and surgical approaches

Classification	Number of cases	Tumor body location	Scope of tumor	Invasion direction	Surgical route (approach)^a^
I: Lateral	10	Same side of the nasopharynx and ethmoid sinus	One side of nasopharynx, ethmoid sinus, medial orbital wall, 1/2 of the external orbital roof and small wing of the sphenoid bone	Intraorbital and intracranial	Orbital-pterional; 2 with TNE
II: Central	14	Center of the skull base (nasopharynx and ethmoid sinus)	Frontal sinus, ethmoid, 1/2 of the internal orbital roof or intraorbital lateral wall and planum sphenoidale	Intracranial	Extended subfrontal; 12 with TNE
III: Extensive	8	Massive, anterior-medial skull base	A combination of I and II	Extensively, nasopharyngeal, intraorbital and intracranial	5 Frontotemporal orbitozygomatic; 3 extended subfrontal; 7 with TNE

Surgical route: a total of 21 cases (2 unilateral, 12 central and 7 extensive) were combined with transnasal endoscopy (TNE)

1. Type I or unilateral (*n* = 10). The main tumor body was located at the same side of the nasopharynx and ethmoid sinus and grew in an intracranial and intraorbital direction. The scope of invasion included one side of the nasopharynx, ethmoid sinus, medial orbital wall, 1/2 of the external orbital roof and small wing of the sphenoid bone (Fig. 1a–c).

**Fig. 1:**
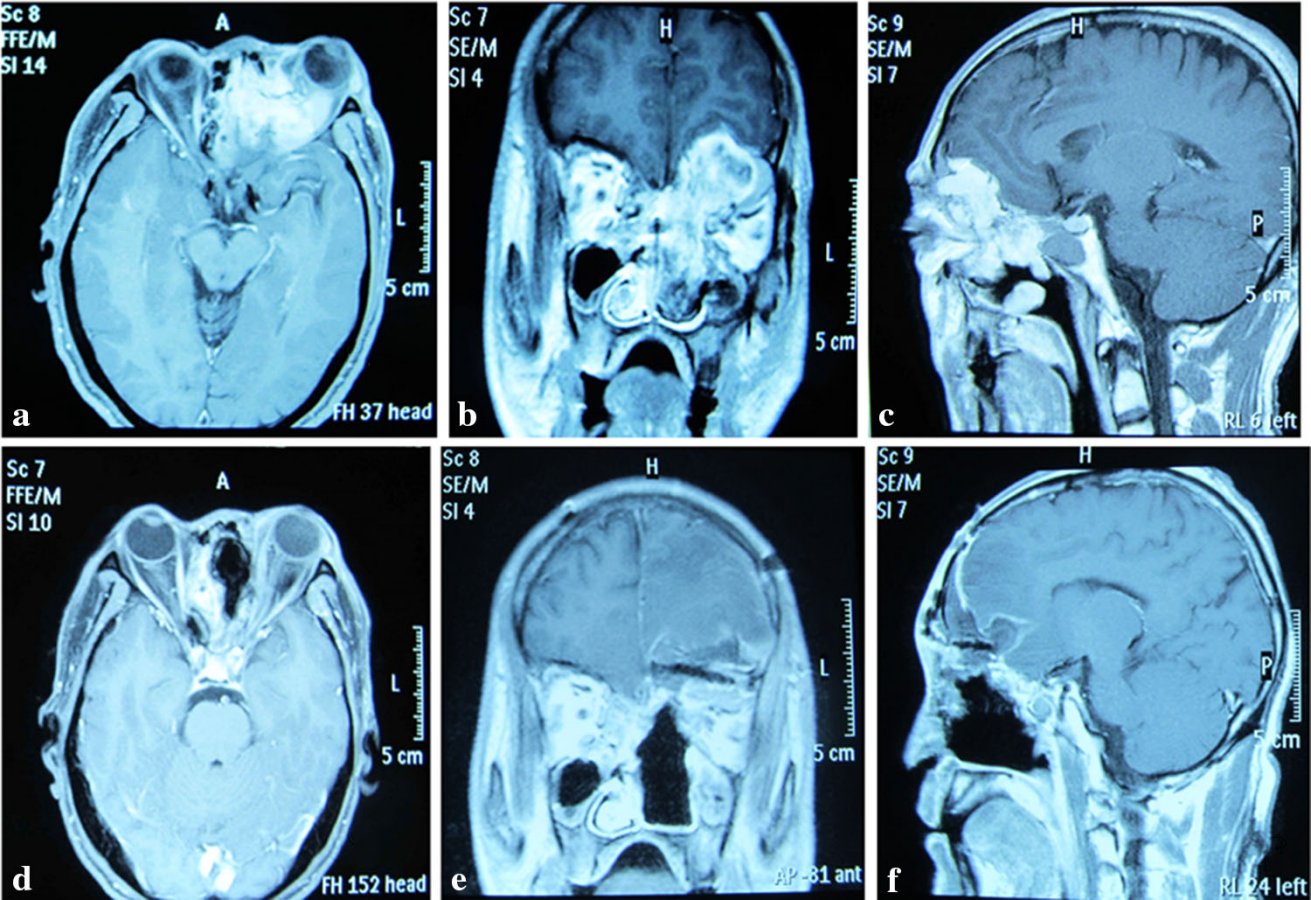
Unilateral lesion. A 48-year-old male presented with a history of progressive exophthalmos accompanied by nasal congestion on the left side. Biopsy of the nasopharyngeal mass revealed a malignant fibrosarcoma. **a**–**c** Preoperative magnetic resonance imaging (MRI) showed a tumor invading the left nasopharynx, ethmoid sinus and left orbit. **d**–**f** MRI images 2 months after surgery show that the tumor was removed completely and the skull base defect was repaired. **e** The orbital-skull defect was reconstructed with a titanium mesh
2. Type II or central (*n* = 14). The main tumor body was located at the center of the skull base (nasopharynx and ethmoid sinus) and grew in an intracranial direction. The scope of invasion included the frontal sinus, ethmoid, 1/2 of the internal orbital roof or intraorbital lateral wall and the planum sphenoidale (Fig. 2a–c).

**Fig. 2:**
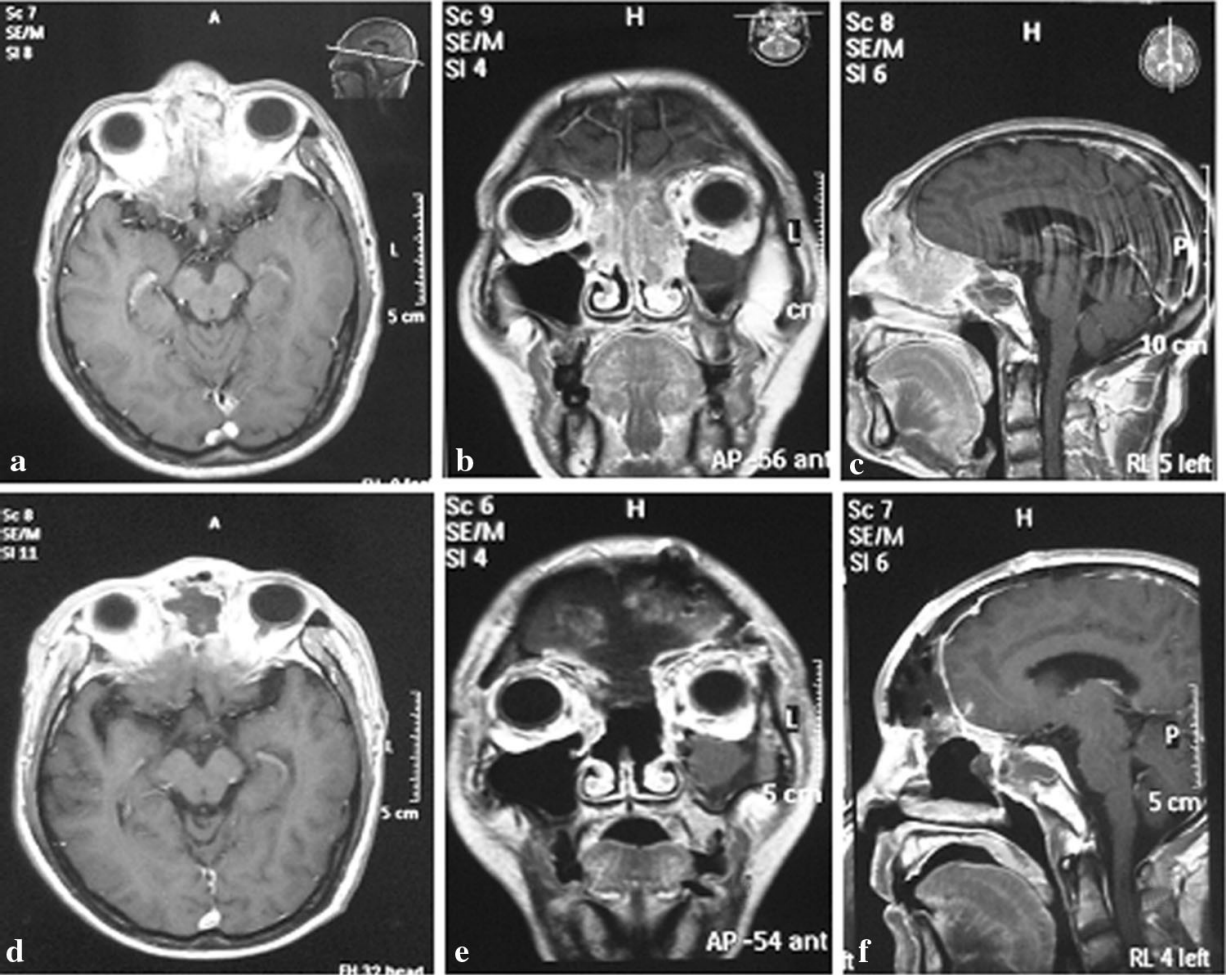
Central lesion. A 54-year-old male presented with a history of headache and nasal congestion. Biopsy revealed an inverted papilloma. **a**–**c** Preoperative magnetic resonance imaging (MRI) showed the tumor invading the frontal–nasal–orbital region, and the frontal bone at the nasion was deformed. **d**–**f** MRI images 3 months after surgery. The tumor and the deformed bone were removed, and the reconstruction of the bone defect was accomplished with a titanium mesh
3. Type III or extensive (*n* = 8). The tumor was massive and located at the anterior-medial skull base, growing extensively in a nasopharyngeal, intraorbital, and intracranial direction. The scope of invasion was a combination of the two previously described (Fig. 3a–c).

**Fig. 3:**
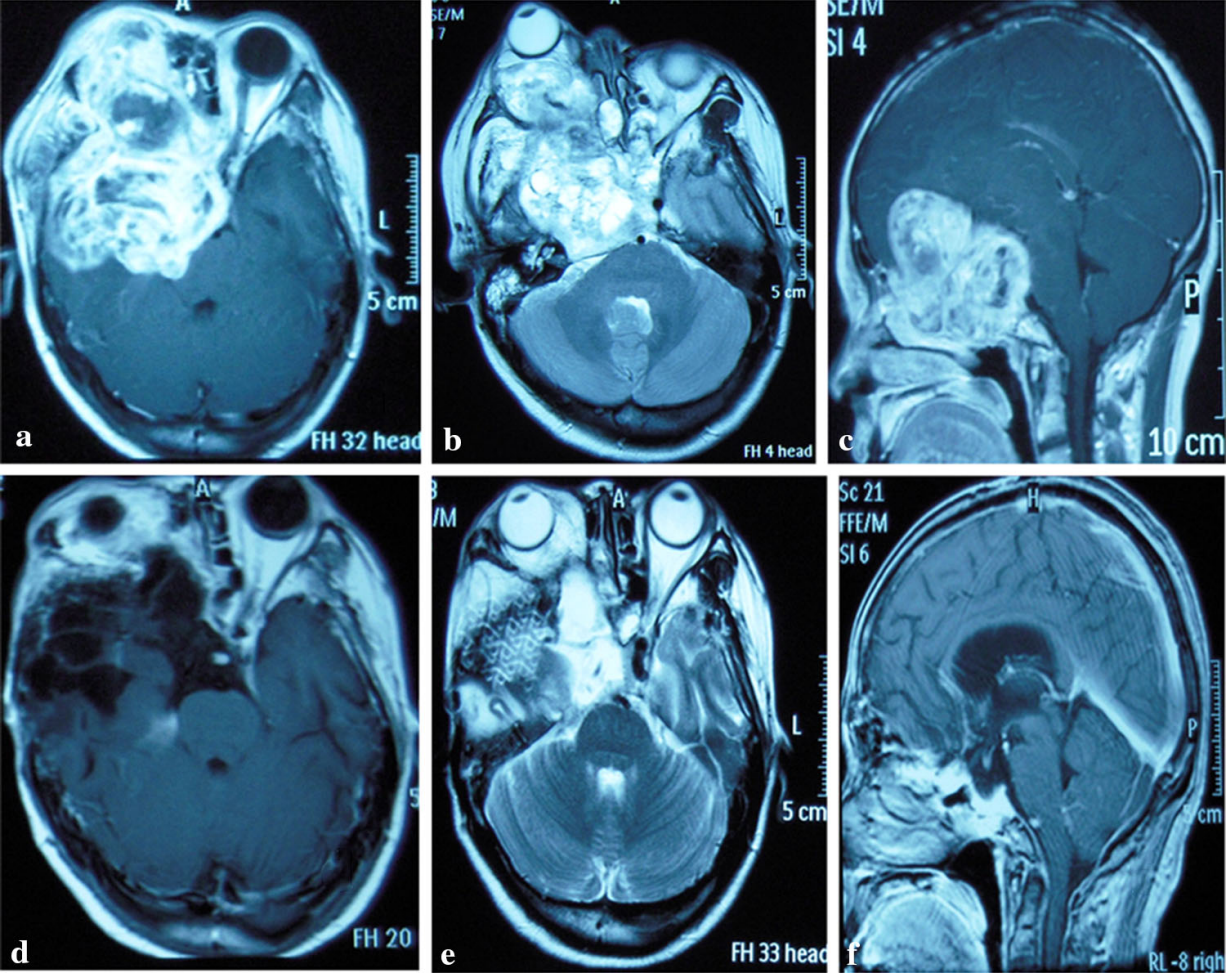
Extensive lesion. A 48-year-old female presented with a history of exophthalmos, headache and vision loss. The diagnosis was a right trigeminal schwannoma. **a**–**c** Preoperative magnetic resonance imaging (MRI) showed extensive invasion of the anterior/middle skull base, orbit, nasopharynx and nasal cavity. **d**–**f** MRI images taken 6 months after surgery show that the tumor was removed completely, and the skull base defect was repaired. **e** A titanium mesh was used to repair the skull base defect

### Treatment protocols

#### Pedicle flap preparation

A pre-surgical multidisciplinary meeting was organized, and an individualized surgical plan was drafted for each patient based on pathological features, imaging data and tumor type. A scalp coronal or semicoronal incision was made at the hairline. Based on the requirements of skull base repair, a skin flap was isolated from the superior or inferior galea aponeurotica to the supraorbital margin [11] and a pedicle flap of 12–14 cm × 6–8 cm in size was prepared for repair of the skull base defect. There were 5 pedicle frontal periosteal grafts, 3 pedicle flaps or pericranial and galea aponeurotica flaps, and 24 pedicle galea aponeurotica superior periosteal flaps.

#### Surgical routes, surgical approaches and tumor resection

The purpose of surgery was to remove as much tumor as possible, whether benign or malignant, with complete resection as the goal. Three surgical routes were adopted based on the tumor classification: orbital-pterional, extended subfrontal and frontotemporal orbitozygomatic. For the orbital-pterional route, the pterion was set as the center; part of the supraorbital margin, the external orbital wall, and part of the orbital roof were removed along with the frontal-temporal flap. The resection range of the residual supraorbital wall and a determination for the need of grinding the superior orbital fissure and optic canal open was based on the tumor location.

For the extended subfrontal route, a unilateral or bilateral frontal flap was prepared, and the lower margin of the flap was kept as close to the supraorbital margin as possible. Using a drill, some parts of the orbital roof, supraorbital margin, nasofrontal fissure and nasal bone were disconnected along with isolation of the frontal flap. Isolation of the dura mater at the epidural region was made as close to the posterior margin of the anterior cranial fossa as possible.

Regarding the frontotemporal orbitozygomatic route, the zygomatic arch was cut using a surgical saw from the upper external to the lower internal regions. A complete flap was constructed from the residual zygoma, supraorbital wall, lateral orbital wall and frontotemporal bone and removed. After flipping open the temporal muscle and removing the external wall of the middle cranial fossa, the upper wall and the external wall of the superior orbital fissure and the upper wall of the optic nerve canal were removed by grinding along the small wing of the sphenoid bone and anterior clinoid process. A pedicle temporalis fascial flap was prepared simultaneously.

In addition to the three routes, nasal endoscopy was used in the combined craniofacial approach for 21 patients with large malignant tumors, extensive invasion or huge benign tumors in which the main body was located outside of the skull for lateral rhinotomy on the basis of the aforementioned approaches.

#### Tumor resection

During surgery, important structures adjacent to the tumors, including the internal carotid artery, cavernous sinus, optic nerve, optic chiasm, pituitary and hypothalamus, were observed under high-power microscopy to avoid injury. Intracranial tumors and tumors in the ethmoid sinus were first removed through the intradural or subdural pathway, followed by resection of tumors in the sphenoid sinus, nasopharynx and intraorbital region. After removing as much tumor as possible in the cranial area, a Karl Storz endoscopic sinus surgery system (wide-angle endoscopes of 0°, 30° and 70° with a diameter of 4 mm and length of 18 cm) was used for further resection. The tumor was isolated from one side of the nasal cavity where the main tumor body resided and then resected from front to back with a stripping tool, metal suction or tumor forceps with a 0° endoscope. Bleeding from the tumor or the anterior or posterior ethmoid artery was electrocoagulated. For tumors invading the maxillary sinus, the ipsilateral uncinate process was removed to open the maxillary sinus and sphenoid. Specific attention was paid to vital structures including the orbital apex, optic nerve and carotid artery. If difficulty was encountered, the bilateral nasal cavity approach was used for resection and hemostasis. Finally, the completeness of excision in the residual cavity of the skull base was examined and repair of the skull base defect was performed aided by nasal endoscopy.

#### Skull base defect repair

For the 20 patients with a skull base defect ≥4 cm in diameter, the dura mater was repaired first with an artificial meningeal patch (*n* = 14), isolated temporalis fascia (*n* = 3) or periosteum (*n* = 3). Then, a previously prepared pedicle flap was inverted backward and tiled on the anterior skull base. The margin of the flap was sutured to the dura mater at the skull base; deep areas which could not be sutured were closed with biological glue. Finally, an appropriately trimmed titanium mesh was placed between the external dura mater and the flap for the repair of the skull base bone defect. In seven patients with a large local residual cavity (diameter >5 cm), the cavity was filled with a previously prepared temporalis muscle flap.

For the 12 patients with a skull base defect diameter <4 cm, the dura mater defect was repaired with an artificial meningeal patch (*n* = 9), isolated temporalis fascia (*n* = 2) or periosteum (*n* = 1), and the pedicle flap was attached to the anterior skull base with sutures or biological glue.

Defects in the external cranial region were closed by muscle paste prepared from temporal muscle retrieved under nasal endoscopy. The paste was made my mashing the retrieved muscle which was then used to fill the defect. The wound was covered with a gelatin sponge and filled/supported by an iodoform strip, both of which were removed 1 week after surgery.

### Postoperative management

A Pan’s drainage tube was placed deep in the surgical field and was removed 3–5 days after surgery. A broad-spectrum antibiotic which easily passes through the blood–brain barrier (ceftriaxone or cefotaxime combined with metronidazole) was administered to all patients for 1 week. Neurotrophic or antiepileptic medications were administered as required. For 14 patients with malignant tumors, radiotherapy alone or combined radiotherapy and chemotherapy was administered. One case with a benign tumor was treated with antiepileptic drugs and antibiotics.

## Results

### Effects of tumor classification and surgical approaches on surgery

The surgical routes were chosen mainly based on the tumor classification: the orbital-pterional route for lateral tumors (type I); the extended subfrontal route for central tumors (type II); for the extensive tumors (type III), the frontotemporal orbitozygomatic route was mainly adopted, except in three cases for which the extended subfrontal route was applied (Table 2). Significant differences in operation time (*P* < 0.001) and blood loss (*P* = 0.004) during operation were observed among different tumor classifications (Table 3) and different surgical routes based on the classification (Table 4). The type I tumors and the corresponding orbital-pterional route had the shortest operation time and lowest blood loss during operation, while the type III tumor and the frontotemporal orbitozygomatic route had the longest operation time and the highest blood loss. These data indicate that the classification method and the corresponding surgical routes exerted significant impact on surgery. In comparison, the tumor size (tumor volume) was not significantly correlated with the operation time (*P* = 0.09) or the amount of blood loss during surgery (*P* = 0.29) (Table S3).

**Table 3:** Effects of tumor classification on operation time and blood loss

	Type of tumor			*F*	*P*
	Lateral	Central	Extensive		
# of cases	10	14	8		
Operation time (h)	4.68 ± 0.8	5.3 ± 0.6	6.1 ± 0.45	13.983	<0.001
Blood loss (ml)	313 ± 134.6	387.9 ± 96.7	510 ± 107.3	6.898	0.004

Statistical difference was determined using one-way ANOVA analysis

**Table 4:** Effects of surgical routes on operation time and blood loss

	Surgical approaches			*F*	*P*
	Orbital-pterional	Extended subfrontal	Frontotemporal orbitozygomatic		
# of cases	10	17	5		
Operation time (h)	4.68 ± 0.8	5.4 ± 0.63	6.3 ± 0.29	10.687	<0.001
Blood loss (ml)	313 ± 134.6	395.9 ± 89.6	556 ± 112.6	8.343	0.001

Statistical difference was determined using one-way ANOVA analysis

### Overall surgical outcomes

Total resection of tumors was achieved in 28/32 patients (87.5 %). There were no perioperative deaths. The average operative time was 5.3 h (range 4–6.6 h), and the average blood loss was 395 ml (range 130–700 ml). Outcomes were determined by combining microscopy observation during surgery and postoperative head CT and MRI (Figs. 1, 2, 3d–f). Of 17 patients with benign tumors, 16 had complete tumor removal and 1 had subtotal resection. Of the 15 patients with malignant tumors, 12 had complete tumor resection and 3 had subtotal resection. The tumor classification, the corresponding surgical approaches and outcomes of individual cases are listed in Supplementary Table 1.

### Clinical improvement after surgery

In all 32 patients, original symptoms were alleviated after surgery, and nasal obstruction, exophthalmos, head distending pain and local craniofacial malformation disappeared. Alleviation of symptoms of diplopia in 12 cases, hypoplasia in 9 cases and visual field defects in 9 cases were observed. No changes were noted in olfactory degeneration and facial paralysis. The quality of life was significantly improved after surgery. The average Karnofsky score evaluated 3 months after surgery was significantly higher (91.25) than that before (81.25) (*P* < 0.001), including 12 cases with a score of 100, 12 cases with 90 and 8 cases with 80.

### Surgical complications

Oculomotor nerve palsy was observed at an early stage in four patients. Two patients had hypoplasia. Rhinorrhea was noted in three patients after the removal of the iodoform strip and resolved after cerebrospinal fluid drainage by lumbar cannulation for 1 week. No severe complications occurred after surgery, such as neurologic impairment, pneumocephalus, meningoencephalocele and intracranial infection. The overall surgical complication rate was 18.8 %.

### Follow-up

All 32 patients were followed up in outpatient clinics, and the average duration of follow-up was 2.99 years (range 6 months to 7 years). Patients with malignant tumors received radiotherapy and chemotherapy as adjuvant treatments. Of the patients with benign tumors, two patients with meningioma relapsed and were treated by gamma knife radiotherapy. Four patients with malignant tumors died, including three who had subtotal resections. One patient with esthesioneuroblastoma died from chemotherapy-induced liver failure at 6 months after surgery. The patients with fibrosarcoma, lacrimal cell carcinoma and transitional cell carcinoma died from intracranial and multiple systemic metastases within 3 years after surgery. As a result, the 3-year survival rate for patients with malignant tumors was 78.6 % (11/14) and the overall 3-year survival rate was 87.5 %. One patient with an invasive pituitary tumor received radiotherapy to prevent a recurrence and had transient cerebrospinal fluid leakage 2 months after surgery, which was resolved after lumbar cannulation and antibiotics.

## Discussion

### Classification of cranial–nasal–orbital communicating tumors

Cranial–nasal–orbital communicating tumors involving the paranasal sinuses, intracranial cavity and orbit are particularly difficult for surgical removal, presenting challenges for skull base reconstruction and a greater risk of post-surgical complications due to a greater range of lesion invasion [2, 3, 9, 14]. Skull base tumors can be classified according to the site of the tumor origin, tumor location (anterior, middle or posterior skull base) and the biological behavior (benign, low-grade or high-grade malignancy) [12]. These methods of classification provided differential guidance for surgical treatments of each specific type of tumor. To date, however, no classification has been specifically aimed to facilitate the selection of surgical approaches, and no classification is available for cranial–nasal–orbital communicating tumors [2, 4, 15]. In this investigation, we have classified 32 cases of communicating tumors (17 benign and 15 malignant) from our institution into three types on the basis of tumor location, extension and direction of invasion: lateral, central and extensive. Below, we discuss the feasibility of this classification in clinical practice.

### Classification facilitates selection of surgical routes

We designed the corresponding surgical approaches to achieve maximum exposure, the shortest surgical distance, the lowest degree of brain tissue stretch, the complete removal of the tumors with minimum operation time and blood loss, and the lowest operative complications. There are three major routes corresponding to the three types of tumors we have classified (Table 2): orbital-pterional, extended subfrontal, and frontotemporal orbitozygomatic corresponding to type I (lateral), II (central) and III (extensive), respectively. However, in three cases of type III, the extended subfrontal approach was applied for consideration of the specific features of the tumors. Overall, the classification and the corresponding surgical approaches exerted significant influence on the operation time and the blood loss during surgery (Tables 3, 4). The analysis validates the feasibility of our classification method, providing substantial guidance for future clinical practice to avoid unnecessarily complicated procedures requiring long operation times, resulting in excess blood loss and possibly tissue damage. As this classification and the corresponding choice of surgical approaches are based on the tumor location, scope of extension and the direction of invasion regardless of the pathological phenotypes or tissue origin, it may also be applicable to other types of skull base tumors.

### Selection of tumor resection for cranial–nasal–orbital communicating tumors

As described above, the selection of surgical approaches was mainly based on the classification of the tumors, as determined by the location of the tumor main body and the extent and direction of invasion/extension. Nevertheless, the pathological data obtained before and during surgery helped determine the extent of surgical resection. The benign tumors were resected along the border as far as possible with the assistance of an endoscope. Malignant tumors were resected as much as possible according to the principle of protecting and preserving the function of the surrounding organs/tissues.

For large tumors with the main tumor body located in the extracranial space and malignant tumors with invasive growth to a wider area, complete exposure is difficult to achieve. The use of the combined craniofacial approach has become conventional and widely used for resection of skull base tumors in a deep location and with a large scope of extension [4, 11, 16], including communicating tumors [17, 18]. The use of transnasal endoscopy has become a standard and it is critical for the success of the craniofacial approach, as it can be used to determine the extent of the tumor and observe for the presence of residual tumor to thoroughly remove the tumor invading the anterior and middle cranial fossa en bloc. Meanwhile, it helps protect important neurovascular structures, reconstruct the skull base and prevent cerebrospinal fluid leakage. The technique of decompression of the optic canal, repair of cerebrospinal rhinorrhea and hypophysectomy with the aid of a transnasal endoscope has been well developed, and the application has been extended to the removal of meningiomas and chordomas [3, 8, 19–21]. However, with the combined transcranial and transnasal approach, the brain tissue is often pulled obviously, resulting in severe cerebral edema after the operation. Therefore, of the 32 cases in our study, 11 patients with the tumor body located mainly in the cranial space were treated by the simple transcranial approach, which was sufficient to fully expose and resect intracranial tumors. The other 21 were treated by the combined craniofacial approach with the aid of transnasal endoscopy. Among them, 2 underwent lateral rhinotomy to remove the massive tumor located in the extracranial region and the other 19 patients underwent extracranial tumor resection through a unilateral or bilateral nasal opening via endoscopy. In addition, the individualized surgical protocol drafted by the multidisciplinary group for each patient is also critical for the success of the surgical treatment. Through these approaches, radical resection was achieved in 87.5 % of the patients (28/32), with a low rate of complications, no perioperative deaths or new neurological defects. The overall surgical outcome is ideal.

### Treatment of post-surgical skull base defects

Various degrees of damage to the dura mater at the skull base and cranial bone are found after removing the cranial–nasal–orbital communicating tumors. A skull base defect results in the communication of brain tissue with the orbit, ethmoid sinus and even nasopharynx, which can lead to severe complications including cerebrospinal fluid leakage, intracranial infection, pneumocephalus and meningoencephalocele [9]. Cerebrospinal fluid leakage occurs in 5.7–30 % of tumors at the midline region of the skull base when treated with the endoscopic transnasal approach [19, 22]. Thus, the reconstruction of the skull base defect has been considered a key in successful skull base tumor surgery [9, 14, 23–26]. Currently, there are no recognized standards for the selection of materials and methods for repairing a skull base defect after surgery. Materials for repair are mainly divided into two major categories: artificial synthetic materials and autologous biologic materials. The former are mainly titanium ethmoid sinus plates and artificial dura mater patches, and the latter includes calvarium and a variety of isolated or vascularized periosteal flaps. The effects of different materials and methods for repairing an anterior skull base defect are not consistent, and the occurrence rate of complications has been reported to range from 0 to 21.9 % [27, 28].

In this study, the following principles were complied with: (1) Dura mater defects of 1 cm × 2 cm or less in size at the skull base were repaired with isolated periosteum and a temporalis fascia flap. For deep regions where sutures could not be applied, a combination of temporal muscle paste and fibrin glue was used. (2) Skull base bone defects with a diameter <4 cm were closed by a pedicle flap on the anterior skull base, which was fixed to the dura mater by sutures or biological glue. For defects with a diameter ≥4 cm or for those that affected the appearance, a pedicle flap was placed on the anterior skull base and sutured or fixed with biological glue, followed by placement of a titanium mesh between the outer dura mater and pedicle flap. The depth of the cavity was filled by an elongated temporalis muscle flap. In the extracranial region, the defect was covered by muscle paste and a gelatin sponge under nasal endoscopy, and supported by an iodoform strip, which was removed 1 week after surgery. Using this repair method, no complications occurred after hospital discharge.

For surgical treatment, the difference between benign and malignant tumors is of less impact, as the surgical strategy was always to remove as much of the lesion as possible with the goal of complete resection. The management methods we have described are applicable to both malignant and benign tumors, and the outcomes of the 32 patients indicate that it is practical for preoperative classification of communicating tumors regardless of their benign or malignant nature.

Our study has limitations. The sample size is small, and our classification did not take into account the difference between benign and malignant tumors. Thus, in future studies, we need to increase the number of cases from multiple institutions. Moreover, preoperative biopsy for pathological diagnosis would be essential for making a more precise classification by considering the difference between benign and malignant tumors.

## Conclusions

We classified 32 cases of cranial–nasal–orbital communicating tumors into three main types according to the tumor body location, the scope of extension and the direction of invasion. The classification facilitated the choice of surgical routes, exerting significant impact on surgical time and the amount of hemorrhage during operation. Combined with individualized strategies developed by a multidisciplinary group and the aid of nasal endoscopy for large and extensive tumors, as well as carefully designed skull base reconstruction, ideal surgical outcomes were achieved, with low complication and morbidity rates, zero perioperative deaths and a high 3-year survival rate of patients with malignant tumors (73.3 %; 11/15). As the simple classification method is based on the general properties of the tumors (location, scope of extension and invading direction) rather than the specific pathological or tissue types, it may also be applicable to other types of skull base tumors.

## Electronic supplementary material

Below is the link to the electronic supplementary material.

Supplementary material 1 (DOC 193 kb)
