# Supplementary material for: A simple classification of cranial–nasal–orbital communicating tumors that facilitate choice of surgical approaches: analysis of a series of 32 cases
Source: Eur Arch Otorhinolaryngol. 2016 Mar 26;273:2239–48. doi: 10.1007/s00405-016-4003-8 (PMC4930795; doi:10.1007/s00405-016-4003-8)
Supplement: Supplementary file 1 — Supplementary material 1 (DOC 193 kb) [file 405_2016_4003_MOESM1_ESM.doc]

**Table S1. Demographic data of the 32 patients**

| | **patient #** | **sex** | **age** | **onset time (months)** | **symptoms and signs** | **tumor type** | **tumor size and range** | **diameter of skull base defect (cm)** | | --- | --- | --- | --- | --- | --- | --- | --- | | 1 | M | 29 | 3 | nasal congestion, headache, diplopia, visual field defect, epilepsy, hyposmia | olfactory neuroblastoma | 6×5.5×6.3cm, NP, ES, FS, prefrontal, BO, OA | 5 | | 2 | M | 17 | 0.5 | epistaxis, nasal congestion, headache, hyposmia | nasopharyngeal angiofibroma | 4×4.5×5.2cm, NP, ES, SS, inferior frontal | 3.8 | | 3 | F | 52 | 21 | right exophthalmos, diplopia, reduced vision, visual field defect, right frontal orbital ridges | meningioma | 4×4.5×3cm, right orbital ball, OA; SF, right NP; ES | 4.2 | | 4 | M | 26 | 9 | Left frontal orbital ridge deformities, numbness，left eye diplopia, reduced vision, proptosis, conjunctival redness | trigeminal schwannoma | 7×6.5×8cm, left frontal orbital, middle cranial fossa, TF, NP | 6.5 | | 5 | M | 65 | 11 | tinnitus, nasal congestion, epistaxis, hyposmia, diplopia, proptosis, reduced vision | undifferentiated carcinoma | 4.5×5×6.3cm, NP, SS, bilateral medial OW, inferior frontal | 4.2 | | 6 | M | 55 | 3 | headache, nasal congestion, diplopia, reduced vision, visual field defect, hyposmia | olfactory neuroblastoma | 5.2×4.5×4.8cm, NP, ES, inferior frontal, BO, OA | 3.8 | | 7 | F | 16 | 7 | right diplopia, reduced vision, exophthalmos | trigeminal schwannoma | 4.2×3×3.8cm, right OA, SF, orbital, NP | 3.8 | | 8 | F | 43 | 4 | right frontal orbital pain, tinnitus, diplopia, epistaxis, nasal congestion, face numbness | squamous cell carcinoma | 6.5×5.5×7cm, NP, SS, ES, prefrontal, BO, right medial SB | 4.6 | | 9 | M | 57 | 26 | nasal congestion, headache, hyposmia, diplopia, visual field defect | olfactory neuroblastoma | 4.2×5.3×5cm, SS, ES, NP, bilateral medial OW | 3.5 | | 10 | F | 30 | 9 | right frontal orbital maxillofacial uplift, proptosis, reduced vision, visual field defect, diplopia | ossifying fibroma | 5×5.7×5.3cm, right maxillary sinus, NP, ES, orbital and OA | 4.1 | | 11 | a | 75 | 16 | nasal congestion, epistaxis, hyposmia, forehead pain | small cell carcinoma | 4.3×4.8×5.6, NP, SS, ES, inferior frontal and bilateral medial orbital | 3.8 | | 12 | M | 61 | 8 | left frontal orbital lumps, ridges and pain, exophthalmos, reduced vision, conjunctival redness | adenoid cystic carcinoma of the lacrimal gland | 5×5.6×5.3cm, left orbital, ES, NP, frontal lobe, TF | 6.2 | | 13 | M | 51 | 13 | nasal congestion, tinnitus, epistaxis, diplopia, hyposmia | squamous cell carcinoma | 6×6×5cm, bilateral ES, SS, medial orbital, prefrontal, NP | 4.5 | | 14 | M | 21 | 2 | nasal congestion, epistaxis, forehead pain, hyposmia | nasopharyngeal angiofibroma | 4×5.2×4.8cm, NP, ES, SS, inferior frontal | 3.5 | | 15 | F | 51 | 5 | headache, nasal congestion, left eye diplopia, reduced vision, visual field defect, proptosis, epilepsy | meningioma | 7×8×10cm, inferior frontal, anterior SB, ES, SS, NP, BO | 5 | | 16 | M | 66 |  | nasal congestion, headache, diplopia, hyposmia | olfactory neuroblastoma | 5×6.5×4.8cm, NP, ES, FS, inferior frontal, BO | 4.2 | | 17 | M | 46 | 7 | left frontal orbital ridges, pain, exophthalmos, diplopia, reduced vision, conjunctival redness | meningioma | 3.8×4×5.3cm, left frontal, OA, orbital and left ES, NP | 3.5 | | 18 | F | 36 | 0.7 | left frontal orbital ridges，pain, exophthalmos, reduced vision, conjunctival redness, epilepsy | malignant fibrosarcoma | 4.5×5.3×4cm, left SF, ES, left orbital, NP | 4.5 | | 19 | M | 44 | 3 | reduced vision, visual field defect, nasal congestion, headache, hyposmia | pituitary tumor | 5×6.2×4cm, NP, SS, ES, sella, inferior frontal | 3.5 | | 20 | F | 56 | 72 | right proptosis, reduced vision，conjunctival redness, right frontal orbital ridge deformities, pain | trigeminal schwannoma | 14.5×8×6cm, right anterior and medial SB, NP, ES, orbital | 7 | | 21 | M | 33 | 3 | tinnitus, diplopia, epistaxis, headache, stuffy nose, hyposmia | squamous cell carcinoma | 4×5.5×5cm, bilateral ES, SS sinus, SF, NP | 3.8 | | 22 | M | 52 | 2 | right frontal orbital ridges, pain, diplopia, reduced vision, visual field defect, exophthalmos, conjunctival redness | malignant fibrosarcoma | 4×3.8×6cm, SF, left orbital, ES, NP | 4 | | 23 | M | 71 | 10 | left diplopia, reduced vision, exophthalmos, left frontotemporal uplift and pain, stuffy nose | meningioma | 6×5.5×7cm, left frontal orbital, NP, ES, TF | 5 | | 24 | M | 19 | 3 | epistaxis, nasal congestion, headache, hyposmia | nasopharyngeal angiofibroma | 4.6×5.8×5.5cm, NP, SS, ES, inferior frontal | 3.7 | | 25 | F | 47 | 4 | nasal congestion, hyposmia, visual field defect, headache, epilepsy | olfactory neuroblastoma | 6×6.5×5cm, bilateral ES, SS, prefrontal, NP | 4.5 | | 26 | F | 49 | 3 | nasal congestion, epistaxis | melanoma | 5.5×6×6.3cm, NP, SS, ES, BO and inferior frontal | 3.8 | | 27 | M | 19 | 13 | right proptosis, reduced vision, conjunctival redness | ossifying fibroma | 3.5×4×4.8, right orbital and orbital roof, inferior frontal, ES, NP | 4.2 | | 28 | F | 37 | 12 | left frontal orbital ridges, pain, diplopia, exophthalmos, hyposmia | trigeminal schwannoma | 4×4.2×5cm, left orbital and ES, NP, SF | 4 | | 29 | M | 42 | 24 | stuffy nose, hyposmia, headache | paraganglioma | 3.3×3.8×4.6cm, NP, ES, bilateral medial OW, SF | 3.3 | | 30 | F | 47 | 3 | reduced vision, headache, binoculus visual field defect, hyposmia, epilepsy | olfactory groove meningioma | 4.5×5.2×5.6cm, BO, NP, ES and inferior frontal | 5.2 | | 31 | F | 74 | 24 | headache, diplopia, proptosis, reduced vision, conjunctival redness, nasal congestion, hyposmia | chordoma | 6×4×5cm, BO, NP, ES, suprasellar | 5.5 | | 32 | M | 64 | 0.7 | stuffy nose, epistaxis, conjunctival redness | inverting papilloma | 4×4.2×5cm, left NP, left orbital, ES, SF | 4 | |
| --- | --- | --- | --- | --- | --- | --- | --- | --- | --- | --- | --- | --- | --- | --- | --- | --- | --- | --- | --- | --- | --- | --- | --- | --- | --- | --- | --- | --- | --- | --- | --- | --- | --- | --- | --- | --- | --- | --- | --- | --- | --- | --- | --- | --- | --- | --- | --- | --- | --- | --- | --- | --- | --- | --- | --- | --- | --- | --- | --- | --- | --- | --- | --- | --- | --- | --- | --- | --- | --- | --- | --- | --- | --- | --- | --- | --- | --- | --- | --- | --- | --- | --- | --- | --- | --- | --- | --- | --- | --- | --- | --- | --- | --- | --- | --- | --- | --- | --- | --- | --- | --- | --- | --- | --- | --- | --- | --- | --- | --- | --- | --- | --- | --- | --- | --- | --- | --- | --- | --- | --- | --- | --- | --- | --- | --- | --- | --- | --- | --- | --- | --- | --- | --- | --- | --- | --- | --- | --- | --- | --- | --- | --- | --- | --- | --- | --- | --- | --- | --- | --- | --- | --- | --- | --- | --- | --- | --- | --- | --- | --- | --- | --- | --- | --- | --- | --- | --- | --- | --- | --- | --- | --- | --- | --- | --- | --- | --- | --- | --- | --- | --- | --- | --- | --- | --- | --- | --- | --- | --- | --- | --- | --- | --- | --- | --- | --- | --- | --- | --- | --- | --- | --- | --- | --- | --- | --- | --- | --- | --- | --- | --- | --- | --- | --- | --- | --- | --- | --- | --- | --- | --- | --- | --- | --- | --- | --- | --- | --- | --- | --- | --- | --- | --- | --- | --- | --- | --- | --- | --- | --- | --- | --- | --- | --- | --- | --- | --- | --- | --- | --- | --- | --- | --- | --- | --- | --- | --- | --- | --- | --- | --- | --- | --- | --- |

Note: ES: ethmoid sinus; SS: sphenoid sinus; FS: frontal sinus; NP: nasopharynx; BO: bilateral orbital; SF: subfrontal; SB: skull base; OA: orbital apex; TF: temporal fossa.

Table S2. Tumor classification, surgical route selections and surgical outcomes

| | # | tumor classification | surgical route | operation time (hr) | blood loss (ml) | follow up | KPS score  BS | KPS score, AS | surgical complication | death |  | | --- | --- | --- | --- | --- | --- | --- | --- | --- | --- | --- | | 1 | Extensive | extensive subfrontal + TNE | 6.5 | 400 | 60 | 80 | 90 |  |  |  | | 2 | Central | extensive subfrontal + TNE | 5.2 | 500 | 60 | 90 | 100 |  |  |  | | 3 | Lateral | orbital-pterional | 4.6 | 380 | 84 | 80 | 90 | oculomotor  nerve palsy | |  | | 4 | Extensive | frontotemporal orbitozygomatic + TNE | 6.5 | 580 | 36 | 80 | 100 |  |  |  | | 5 | Central | extensive subfrontal + TNE | 5 | 350 | 26 | 80 | 90 |  | 26 months after surgery, intracranial and systemic metastases |  | | 6 | Extensive | extensive subfrontal + TNE | 5.5 | 450 | 48 | 80 | 90 |  |  |  | | 7 | Lateral | orbital-pterional | 4.2 | 130 | 60 | 80 | 100 |  |  |  | | 8 | Extensive | frontotemporal orbitozygomatic + TNE | 6.6 | 500 | 24 | 80 | 90 |  |  |  | | 9 | Central | extensive subfrontal + TNE | 5.8 | 400 | 28 | 80 | 90 |  |  |  | | 10 | Lateral | orbital-pterional + TNE | 4.8 | 420 | 36 | 80 | 100 |  |  |  | | 11 | Central | extensive subfrontal + TNE | 5.6 | 500 | 14 | 70 | 90 |  |  |  | | 12 | Lateral | orbital-pterional | 6.2 | 400 | 34 | 80 | 80 | oculomotor nerve palsy | 34 months after surgery, intracranial and systemic metastases |  | | 13 | Central | extensive subfrontal + TNE | 6.3 | 420 | 18 | 80 | 90 |  |  |  | | 14 | Central | extensive subfrontal + TNE | 4.5 | 450 | 36 | 90 | 100 |  |  |  | | 15 | Extensive | frontotemporal orbitozygomatic + TNE | 6 | 700 | 72 | 70 | 90 | Oculomotor  nerve palsy,  reduced vision | |  | | 16 | Central | extensive subfrontal + TNE | 5.5 | 450 | 6 | 80 | 90 |  | 6 months after chemotherapy-induced liver failure |  | | 17 | Lateral | orbital-pterional | 4 | 200 | 36 | 90 | 100 |  |  |  | | 18 | Lateral | orbital-pterional | 6 | 500 | 29 | 70 | 90 |  | 36 months after surgery, systemic metastases |  | | 19 | Central | extensive subfrontal + TNE | 5.8 | 250 | 33 | 80 | 90 | CSF  leaking | |  | | 20 | Extensive | frontotemporal orbitozygomatic + TNE | 6 | 400 | 48 | 80 | 90 |  | |  | | 21 | Central | extensive subfrontal | 5 | 380 | 22 | 90 | 90 |  | |  | | 22 | Lateral | orbital-pterional | 4.5 | 300 | 24 | 80 | 80 | oculomotor  nerve palsy,  reduced vision | |  | | 23 | Extensive | frontotemporal orbitozygomatic | 6.5 | 600 | 36 | 80 | 90 |  | |  | | 24 | Central | extensive subfrontal + TNE | 5.8 | 500 | 28 | 90 | 100 |  | |  | | 25 | Extensive | extensive subfrontal + TNE | 5.5 | 450 | 24 | 80 | 100 |  | |  | | 26 | Central | extensive subfrontal + TNE | 5 | 400 | 16 | 90 | 100 |  | |  | | 27 | Lateral | orbital-pterional + TNE | 4 | 200 | 15 | 80 | 100 |  | |  | | 28 | Lateral | orbital-pterional | 4.5 | 150 | 22 | 90 | 100 |  | |  | | 29 | Central | extensive subfrontal | 4.15 | 200 | 12 | 90 | 100 |  | |  | | 30 | Central | extensive subfrontal + TNE | 4.5 | 380 | 12 | 80 | 100 |  | |  | | 31 | Central | extensive subfrontal + TNE | 5.5 | 250 | 11 | 70 | 90 |  | |  | | 32 | Lateral | orbital-pterional | 4 | 450 | 6 | 80 | 100 |  | |  | |
| --- | --- | --- | --- | --- | --- | --- | --- | --- | --- | --- | --- | --- | --- | --- | --- | --- | --- | --- | --- | --- | --- | --- | --- | --- | --- | --- | --- | --- | --- | --- | --- | --- | --- | --- | --- | --- | --- | --- | --- | --- | --- | --- | --- | --- | --- | --- | --- | --- | --- | --- | --- | --- | --- | --- | --- | --- | --- | --- | --- | --- | --- | --- | --- | --- | --- | --- | --- | --- | --- | --- | --- | --- | --- | --- | --- | --- | --- | --- | --- | --- | --- | --- | --- | --- | --- | --- | --- | --- | --- | --- | --- | --- | --- | --- | --- | --- | --- | --- | --- | --- | --- | --- | --- | --- | --- | --- | --- | --- | --- | --- | --- | --- | --- | --- | --- | --- | --- | --- | --- | --- | --- | --- | --- | --- | --- | --- | --- | --- | --- | --- | --- | --- | --- | --- | --- | --- | --- | --- | --- | --- | --- | --- | --- | --- | --- | --- | --- | --- | --- | --- | --- | --- | --- | --- | --- | --- | --- | --- | --- | --- | --- | --- | --- | --- | --- | --- | --- | --- | --- | --- | --- | --- | --- | --- | --- | --- | --- | --- | --- | --- | --- | --- | --- | --- | --- | --- | --- | --- | --- | --- | --- | --- | --- | --- | --- | --- | --- | --- | --- | --- | --- | --- | --- | --- | --- | --- | --- | --- | --- | --- | --- | --- | --- | --- | --- | --- | --- | --- | --- | --- | --- | --- | --- | --- | --- | --- | --- | --- | --- | --- | --- | --- | --- | --- | --- | --- | --- | --- | --- | --- | --- | --- | --- | --- | --- | --- | --- | --- | --- | --- | --- | --- | --- | --- | --- | --- | --- | --- | --- | --- | --- | --- | --- | --- | --- | --- | --- | --- | --- | --- | --- | --- | --- | --- | --- | --- | --- | --- | --- | --- | --- | --- | --- | --- | --- | --- | --- | --- | --- | --- | --- | --- | --- | --- | --- | --- | --- | --- | --- | --- | --- | --- | --- | --- | --- | --- | --- | --- | --- | --- | --- | --- | --- | --- | --- | --- | --- | --- | --- | --- | --- | --- | --- | --- | --- | --- | --- | --- | --- | --- | --- | --- | --- | --- | --- | --- | --- | --- | --- | --- | --- | --- | --- | --- | --- | --- | --- | --- | --- | --- | --- | --- | --- | --- | --- | --- | --- | --- | --- | --- | --- | --- | --- |

#: patient number; TNE: transnasal endoscopy; KPS score: Karnofsky score; BS: before surgery; AS: after surgery.

Table S3: Comparison of tumor size and tumor location with tumor type, surgical route, operation time and blood loss during surgery

| **#** | **tumor size (cm)** | **Tumor volume (cm^3^)** | **operation time (hr)** | **blood loss (ml)** | **surgical approach** | **type of tumor** | **TNE** |
| --- | --- | --- | --- | --- | --- | --- | --- |
| 7 | 4.2×3×3.8 | 47.9 | 5.8 | 400 | extensive subfrontal + TNE | Central | TNE |
| 29 | 3.3×3.8×4.6 | 57.7 | 4.2 | 130 | orbital-pterional | Lateral | TNE |
| 27 | 3.5×4×4.8 | 67.2 | 4.5 | 150 | orbital-pterional | Lateral | No |
| 17 | 3.8×4×5.3 | 80.6 | 5.5 | 450 | extensive subfrontal + TNE | Extensive | TNE |
| 28 | 4×4.2×5 | 84.0 | 4 | 450 | orbital-pterional | Lateral | No |
| 32 | 4×4.2×5 | 84.0 | 4.8 | 420 | orbital-pterional + TNE | Lateral | TNE |
| 22 | 4×3.8×6 | 91.2 | 6 | 400 | frontotemporal orbitozygomatic + TNE | Extensive | TNE |
| 2 | 4×4.5×5.2 | 93.6 | 4.15 | 200 | extensive subfrontal | Central | No |
| 18 | 4.5×5.3×4 | 95.4 | 6.5 | 600 | frontotemporal orbitozygomatic | Extensive | No |
| 14 | 4×5.2×4.8 | 99.8 | 4.5 | 380 | extensive subfrontal + TNE | Central | TNE |
| 21 | 4×5.5×5 | 110.0 | 6 | 700 | frontotemporal orbitozygomatic + TNE | Extensive | TNE |
| 9 | 4.2×5.3×5 | 111.3 | 6.3 | 420 | extensive subfrontal + TNE | Central | TNE |
| 6 | 5.2×4.5×4.8 | 112.3 | 5 | 350 | extensive subfrontal + TNE | Central | TNE |
| 11 | 4.3×4.8×5.6 | 115.6 | 5.5 | 450 | extensive subfrontal + TNE | Central | TNE |
| 31 | 6×4×5 | 120.0 | 4 | 200 | orbital-pterional + TNE | Lateral | TNE |
| 19 | 5×6.2×4 | 124.0 | 6.6 | 500 | frontotemporal orbitozygomatic + TNE | Extensive | No |
| 30 | 4.5×5.2×5.6 | 131.0 | 4.6 | 380 | orbital-pterional | Lateral | No |
| 3 | 4×6×5.5 | 132.0 | 5.8 | 500 | extensive subfrontal + TNE | Central | TNE |
| 24 | 4.6×5.8×5.5 | 137.2 | 4 | 200 | orbital-pterional | Lateral | No |
| 5 | 4.5×5×6.3 | 141.8 | 5.2 | 500 | extensive subfrontal + TNE | Central | TNE |
| 12 | 5×5.6×5.3 | 148.4 | 5.8 | 250 | extensive subfrontal + TNE | Central | TNE |
| 10 | 5×5.7×5.3 | 151.1 | 4.5 | 450 | extensive subfrontal + TNE | Central | TNE |
| 16 | 5×6.5×4.8 | 156.0 | 6.5 | 400 | extensive subfrontal + TNE | Extensive | TNE |
| 13 | 6×6×5 | 180.0 | 5 | 400 | extensive subfrontal + TNE | Central | TNE |
| 25 | 6×6.5×5 | 195.0 | 6 | 500 | orbital-pterional | Lateral | No |
| 1 | 6×5.5×6.3 | 207.9 | 5 | 380 | extensive subfrontal | Central | No |
| 26 | 5.5×6×6.3 | 207.9 | 4.5 | 300 | orbital-pterional | Lateral | No |
| 4 | 7×6.5×8 | 227.5 | 5.5 | 450 | extensive subfrontal + TNE | Extensive | TNE |
| 23 | 6×5.5×7 | 231.0 | 6.2 | 400 | orbital-pterional | Lateral | No |
| 8 | 6.5×5.5×7 | 250.3 | 5.6 | 500 | extensive subfrontal + TNE | Central | TNE |
| 15 | 7×8×10 | 560.0 | 5.5 | 250 | extensive subfrontal + TNE | Central | TNE |
| 20 | 14.5×8×6 | 1044.0 | 6.5 | 580 | frontotemporal orbitozygomatic + TNE | Extensive | TNE |

Note: correlation coefficient: 0.3 between tumor volume and operation time (P = 0.09); 0.19 between tumor volume and blood loss during surgery (P = 0.29).

Tumor classification and choice of surgical approaches are not related to tumor size. TNE: transnasal endoscopy

Table S4 Radiology findings: tumor body location and extension/invasion

| **#** | **tumor main body location** | **tumor extension/invasion** | **additional features** |
| --- | --- | --- | --- |
| 1 | middle of anterior SB, | NP; ES; FS; BO; bilateral OA; middle cranial fossa; FL through SB dura | bilateral ON, OC, pituitary and PS pressed and shifted; border unclear |
| 2 | NP, middle | ES; SS; SF | bilateral OW pressed and damaged |
| 3 | right side, anterior SB, | RO; OA; SF; right CS and NP | Right ON unclear; the right ICA significantly pressed |
| 4 | border of left anterior and middle SB | Partial tumor cysts; LO; left side of the saddle and the left TF | border clear; left ON unclear; left eye, left CA and left cavernous obvious pressed and shifted; left zygomatic ridges |
| 5 | NP and SS, | Bilateral medial OW and SF | Bilateral ON, bilateral OC, pituitary and ICA pressed and shifted; border unclear |
| 6 | ES and SF sinus | NP; BO and OA | bilateral ON, OC, pituitary and PS obviously pressed; border unclear |
| 7 | Right OA and middle SB | right SF; OA and NP | border clear; ICA pressed |
| 8 | NP, SS | ES; bilateral frontal slope; BO; the slope and the middle SB | CA wrapped; pituitary and bilateral ON and OC pressed and unclear |
| 9 | SS and ES | NP and FS, and BO wall | right orbital ridge and orbital bone damage and hyperplasia |
| 10 | right ES, medial and top orbital | NP; OR and right SF | border unclear |
| 11 | SS and ES | NP; SF and BO |  |
| 12 | Left orbit | ES; NP; left FL through the OR; TF and subcutaneous |  |
| 13 | NP, bilateral ES and SS | BO; bottom of FL | bilateral FL, cavernous and ICA obvious pressed and shifted; border unclear; bilateral ON and pituitary unclear |
| 14 | NP, ES | BO; SS; intracranial to SF | bilateral OC and pituitary pressed |
| 15 | SF and left anterior SB | Frontal bone; ES; LO and NP; OA and OR; left middle SB | Bilateral CA pressed and partially surrounded; pituitary and OC unclear |
| 16 | NP, ES | Bilateral SF; bilateral FS; BO; pituitary and the optic chiasm | ON pressed obviously; border unclear |
| 17 | left OR; left SF | left ES and NP; |  |
| 18 | left anterior SB | skin; eyebrow; left ES; left orbit and NP | pressed bilateral optic nerve, ICA and OC; |
| 19 | Sella | SS and NP; pituitary and hypothalamus; CS | bilateral ON and ICA pressed |
| 20 | right anterior and middle SB | NP; ES; SS and the LO; right TF and pterygopalatine fossa; | ICA and cavernous unclear |
| 21 | central anterior SB, | NP; BF; BO | Bilateral ON, OC and ICA pressed and shifted |
| 22 | right anterior SB and RO | right ES and NP; eyebrow and FS; OW; OA | left CA pressed |
| 23 | Left anterior SB, | ES and NP; the skull and TF; the right ES and OA; | left CA pressed |
| 24 | central anterior SB, | NP; bilateral FL; BO | Bilateral ON, OC and ICA pressed and shifted |
| 25 | central anterior SB, | NP; bilateral SF; bilateral medial OW; SS | Bilateral ON and OC pressed and shifted; pituitary unclear |
| 26 | central anterior SB, | nose and FS; NP and the SS; FL; BO; OC and pituitary | |
| 27 | Front right side of the SB, | eyebrow; OA; midline crest; epidural; RR, ES and NP | border unclear |
| 28 | Junction of the left anterior and middle SB | superior TS; left ES and OA; LO and NP | border relatively clear |
| 29 | central anterior SB, | epidura; NP; BO | border relatively clear; ON and bilateral medial OW pressed and damaged |
| 30 | central anterior SB, | bilateral FL; NP; | border less clear, ON and OC pressed |
| 31 | Sella and the petroclivus | nose and FS; BO; NP; | border unclear; surrounded bilateral OC; hypothalamus pressed and shift |
| 32 | left anterior SB, | epidural cranial cavity; LO and left NP; orbital rim | border less clear |

Abbreviations: ES: ethmoid sinus; FS: frontal sinus; SS: sphenoid sinus: NP: nasopharynx; BO: bilateral orbital; OR: orbital roof; OA: orbital apex; OW: orbital wall; LO: left orbital; RO: right orbital; CS: cavernous sinus; ON: optic nerve; OC: optic chiasm; CA: carotid artery; ICA: internal carotid artery; TF: temporal fossa; SB: skull base; SF: subfrontal; PS: pituitary stalk.

**Table S5. Summary of tumor body locations**

| **Tumor body locations** |  |  | **number of patients** |
| --- | --- | --- | --- |
| skull base (SB) |  |  |  |
| anterior SB |  |  |  |
| left |  |  | 3 |
| right |  |  | 2 |
| central |  |  | 7 |
| anterior and middle SB |  |  | 1 |
| junction of anterior and middle SB | |  | 2 |
| sella + petroclivus |  |  | 2 |
| skull base + orbital-nasal sinus | |  | 3 |
| orbital, nasopharynx, and sinuses | |  | 12 |
| Total |  |  | 32 |
